# Supplementary material for: Chromothripsis during telomere crisis is independent of NHEJ, and consistent with a replicative origin
Source: Genome Res. 2019 May;29(5):737–49. doi: 10.1101/gr.240705.118 (PMC6499312; doi:10.1101/gr.240705.118)
Supplement: Supplemental Material [file supp_gr.240705.118_Supplemental_file_1.zip › contigs/annotated_contigs/DB116/contig.2.DB116_length_784_mean_cov_26.3290816327.docx]

**DB116_length_784_mean_cov_26.3290816327**

```
GCCCGACCGACCCAGCCCTTAGAGCCAATCCTTATCCCGAAGTTACGGATCCGGCTTGCCGACTTCCCTTACCTACATTGTTCCAACAT
 >chrUn_gl000220:115502-116260 - E=0e+00
GCCAGAGGCTGTTCACCTTGGAGACCTGCTGCGGATATGGGTACGGCCCGGCGCGAGATTTACACCCTCTCCCCCGGATTTTCAAGGGC

CAGCGAGAGCTCACCGGACGCCGCCGGAACCGCGACGCTTTCCAAGGCACGGGCCCCTCTCTCGGGGCGAACCCATTCCAGGGCGCCCT

GCCCTTCACAAAGAAAAGAGAACTCTCCCCGGGGCTCCCGCCGGCTTCTCCGGGATCGGTCGCGTTACCGCACTGGACGCCTCGCGGCG

CCCATCTCCGCCACTCCGGATTCGGGGATCTGAACCCGACTCCCTTTCGATCGGCCGAGGGCAACGGAGGCCATCGCCCGTCCCTTCGG

AACGGCGCTCGCCCATCTCTCAGGACCGACTGACCCATGTTCAACTGCTGTTCACATGGAACCCTTCTCCACTTCGGCCTTCAAAGTTC

TCGTTTGAATATTTGCTACTACCACCAAGATCTGCACCTGCGGCGGCTCCACCCGGGCCCGCGCCCTAGGCTTCAAGGCTCACCGCAGC

GGCCCTCCTACTCGTCGCGGCGTAGCGTCCGCGGGGCTCCGGGGGCGGGGAGCGGGGCGTGGGCGGGAGGAGGGGAGGAGGCGTGGGGG

GGGCGGGGGAAGGACCCCACACCCCCGCCGCCGCCGCCGCCGC|TC|CCGTCCACTCTCGACTGCCGGC|GACGG                                              >chr4:70296618-70296640 +
```
